# Impact of COVID-19 lockdown on mental health in Germany: longitudinal observation of different mental health trajectories and protective factors

**DOI:** 10.1038/s41398-021-01508-2

**Published:** 2021-07-17

**Authors:** K. F. Ahrens, R. J. Neumann, B. Kollmann, J. Brokelmann, N. M. von Werthern, A. Malyshau, D. Weichert, B. Lutz, C. J. Fiebach, M. Wessa, R. Kalisch, M. M. Plichta, K. Lieb, O. Tüscher, A. Reif

**Affiliations:** 1 Department of Psychiatry, Psychosomatic Medicine and Psychotherapy, University Hospital Frankfurt, Frankfurt, Germany; 2 Department of Psychiatry and Psychotherapy, University Medical Center Mainz, Mainz, Germany; 3 Leibniz Institute for Resilience Research (LIR) gGmbH, Mainz, Germany; 4 Institute of Physiological Chemistry, University Medical Center Mainz, Mainz, Germany; 5 Department of Psychology, Goethe University Frankfurt, Frankfurt, Germany; 6 Brain Imaging Center, Goethe University, Frankfurt, Germany; 7 Department of Clinical Psychology and Neuropsychology, Institute for Psychology, Johannes Gutenberg University Mainz, Mainz, Germany; 8 Neuroimaging Center (NIC), Focus Program Translational Neuroscience (FTN), University Medical Center Mainz, Mainz, Germany

**Keywords:** Scientific community, Human behaviour

## Abstract

The COVID-19 pandemic and resulting measures can be regarded as a global stressor. Cross-sectional studies showed rather negative impacts on people’s mental health, while longitudinal studies considering pre-lockdown data are still scarce. The present study investigated the impact of COVID-19 related lockdown measures in a longitudinal German sample, assessed since 2017. During lockdown, 523 participants completed additional weekly online questionnaires on e.g., mental health, COVID-19-related and general stressor exposure. Predictors for and distinct trajectories of mental health outcomes were determined, using multilevel models and latent growth mixture models, respectively. Positive pandemic appraisal, social support, and adaptive cognitive emotion regulation were positively, whereas perceived stress, daily hassles, and feeling lonely negatively related to mental health outcomes in the entire sample. Three subgroups (“recovered,” 9.0%; “resilient,” 82.6%; “delayed dysfunction,” 8.4%) with different mental health responses to initial lockdown measures were identified. Subgroups differed in perceived stress and COVID-19-specific positive appraisal. Although most participants remained mentally healthy, as observed in the resilient group, we also observed inter-individual differences. Participants’ psychological state deteriorated over time in the delayed dysfunction group, putting them at risk for mental disorder development. Consequently, health services should especially identify and allocate resources to vulnerable individuals.

## Introduction

The appearance of coronavirus disease 2019 (COVID-19), caused by severe acute respiratory syndrome (SARS) coronavirus 2, was described by the World Health Organization on 31st of January 2020 as a a public health emergency of international concern. The rapid spread of the virus forced many governments around the world to issue measures, such as lockdowns, to avoid further spreading. As these measures affect a large majority of people around the globe, they can be viewed as ubiquitous stressors, likely influencing people’s mental health. Earlier reviews on the effects of previous virus outbreaks, other than COVID-19, on mental health showed overall negative psychosocial and psychological consequences [1, 2]. First representative population-based studies on the immediate psychological responses of the lockdown due to COVID-19 in China found a moderate-to-severe psychological impact in more than half of the respondents; one-third of the respondents reported moderate-to-severe anxiety and one-fifth suffered from depressive symptoms [3]. Further, nationwide investigations on the mental health status of the general public since the beginning of the pandemic in countries such as Spain, Italy, Iran, USA, Turkey, Nepal, or Denmark consistently revealed lower psychological well-being and higher rates of negative mental health outcomes, such as higher rates of loneliness, anxiety, and depressive symptoms [4, 5].

Besides these cross-sectional findings, a recent review by Vindegaard and Benros [6] addressed longitudinal changes in the prevalence of mental health problems. For the general public, they conclude that mental health is affected, and that especially anxiety and depression symptoms increased compared to before COVID-19. Interestingly, a recent longitudinal study in a case–control cohort of people with depressive, anxiety, or obsessive-compulsive disorders and a control group without psychiatric disorders found that especially people without pre-existing psychiatric disorders showed an increase in symptoms during the COVID-19 pandemic [7]. Contrastingly, an ongoing longitudinal panel study in Germany, the German Socio-Economic Panel (SOEP), indicates that participant’s well-being and overall mental health remained largely unchanged, at least within the first 6 weeks after lockdown, except for an increase of loneliness [8]. Another prospective study, comparing psychopathological symptoms right before and post outbreak, could show that psychopathological symptoms did not change for the majority of the participants during the pandemic, whereas 10% of the participants experienced increased symptomatology [9]. Overall, these studies suggest that the global COVID-19 pandemic and the related changes in everyday life mostly worsen mental health in different societies investigated. However, at a closer look, although a relevant rate of individuals seems particularly at risk for a worse mental health outcome, others seem mainly unaffected.

Various studies have investigated predictors for mental health outcomes due to the lockdown, with an emphasis on factors such as demographic and lifestyle variables. Factors frequently reported to be associated with higher risk of mental dysfunctions were younger age, female sex, living without a partner, the presence of physical or psychiatric illnesses, lower education level, low income or unemployment, or employment in the health care system [4, 10–15]. Although the relationship between general demographic variables and risk of mental health problems during the pandemic has been investigated in depth over the last couple of months, results about specific psychologically straining stressors or psychological coping strategies and their effects in provoking mental health issues are rather inconclusive. Some studies investigated potential variables, such as little perceived social support, which unsurprisingly led to more stress, anxiety, or depressive symptoms [16, 17]. Also, negative coping strategies have been found to be negatively related to mental health [15, 17]. However, others failed to find significant protective effects of problem-focused coping (e.g., planning) and positive coping strategies (e.g., positive reframing) for depression, anxiety, and stress during lockdown due to the COVID-19 pandemic, which underlines the need for more research in this field [18].

Notably, most of the recent studies on predictors and the effects of the COVID-19 pandemic on mental health relied on cross-sectional data. Longitudinal studies of the general population that allow for benchmark comparisons of mental health outcomes are scarce and either rely on surveys that were conducted several years ago or/and mainly compare these data to a single time point during lockdown measures (e.g., see refs. [8, 9, 19, 20]) but did not show fluctuations in participant’s mental health over time. One recent study investigated health trajectories for depression and anxiety symptoms week by week, following the introduction of lockdown in England. They found high levels of symptoms in the early stages of the lockdown and reported a rapid decline across the following 20 weeks [21]. Taken together, integrating pre-lockdown data with health trajectories over time has rarely been investigated so far. Observing mental health outcomes over a longer period of time may, however, be very elucidating, as measures such as lockdowns were dynamically issued and relaxed again, possibly affecting the same individual in different ways at different time points. Further, people’s mental health status before the lockdown might determine the trajectory of mental health outcomes during the lockdown. Numerous studies indicate that measures, such as physical distancing, have a more detrimental effect on already vulnerable persons than on persons with good mental health [4, 6]. This would result in heterogeneous trajectories and outcomes of mental health due to the same stressor, as recently assumed by Mancini [22]. Therefore, investigating participants who were deep-phenotyped prior to lockdown measures would be beneficial.

Data presented here are from a subsample (*n* = 523) of participants from the large-scale LOngitudinal Resilience Assessment (LORA) study, who volunteered to participate in additional weekly online measurements from April 2020 onward. The LORA study has been ongoing since 2017 and entails *N* = 1191 deep-phenotyped healthy participants, which are monitored regularly since their baseline inclusion [23]. In an earlier exploratory investigation in the aforementioned subsample, we found an overall reduction of experienced daily hassles (DHs) and an accompanying average improvement in mental health outcomes, measured using the general health questionnaire (GHQ-28 [24, 25]), when the last individual measuring time point prior to the lockdown and the mental health outcome 8 weeks into the lockdown were compared. On a closer look, we identified three distinct subgroups among our sample, which differed in their mental health trajectories over time [26]. The goal of the present exploratory work was to investigate the impact of the lockdown in Germany onto participant’s mental health in greater detail by scrutinizing the temporal dynamics of and factors for the different mental health trajectories observed. Moreover, possible protective mechanisms against mental health deterioration were investigated. This will aid to gain a more differentiated understanding of the lockdown’s impact on mental health outcomes and may contribute to the development of specific interventions.

## Methods

### Study design

The applied study design is an extension of the prospective longitudinal cohort study called LORA, ongoing since February 2017 in Germany. The LORA study investigates mentally healthy participants between 18 and 50 years of age at study entry, who are assessed every 3 months with respect to major life events, micro-stressors through the assessment of DHs, and mental health status (primary outcome: GHQ-28) with an online monitoring system [23]. Notably, participants were interviewed for mental disorders at study entry. For further details on the LORA study design, see Chmitorz et al. [23].

For the current study, the regular sampling rate of 3 months, at which a stressor and mental health monitoring was performed, was increased immediately after lockdown, so that participants were assessed once a week. Besides socio-demographic information (with particular relevance for the pandemic: employment status, size of household, occupation with pandemic and its effects on society, and whether or not participants were affected by the pandemic themselves), the survey includes mental health questionnaires, as well as assessments of stressors and resilience factors as proposed in existing literature [27, 28].

**Fig. 1:**
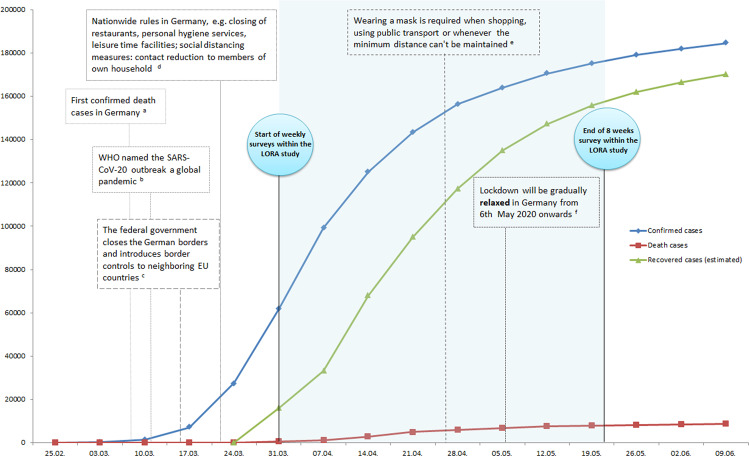
Timing of data acquisition relative to the development of the pandemic in Germany. For footnotes, see the references, ^a^[61], ^b^[62], ^c^[63], ^d^[64], ^e^[65], and ^f^[51].

The increased sampling rate was planned to last for the time of official contact restrictions and a certain time beyond that. Assessments started on the 31st of March and lasted until late May, i.e., briefly before restrictions were relaxed and infection rates plateaued (see Fig. 1 for the timing of the study relative to the development of the pandemic in Germany). Of note, lockdown measures in Germany mandated physical distancing measures, but no forced full lockdown in the form of protracted curfews (see also Fig. 1). It took participants roughly 45 min to fill in the weekly questionnaires, assessed in an online system. Participants were given 3 days each week to fill in the questionnaires to keep the time of assessment similar between subjects and across sites. They were rewarded with 10€ per week for participation. Ethical approval for the increased sampling rate and additional questionnaires, specifically related to the impact of COVID-19, was obtained from the Ethical Review Boards of Mainz and Frankfurt, respectively.

### Participants

All participants of the ongoing LORA study were asked to participate in the weekly assessments on a voluntary basis. For initial inclusion and exclusion criteria, see Chmitorz et al. [23]. Participants could withdraw their consent at any time; they were excluded from participation in the present sub-study after four consecutively missed assessments.

Here, *N* = 523 participants (68.6% female) gave written informed consent electronically via e-mail (for sample description see, Supplementary Table 1 and ref. [26]). In week 8, the subsample comprised *n* = 451 participating subjects (37.9% of the LORA sample), resulting in an attrition rate of 13.8%. This sample did not differ from the full sample of the LORA study with respect to socio-demographic variables, mental dysfunctions, or lifetime life events. All participants were physically and mentally healthy at baseline assessment of the LORA study.

### Mental health outcomes

Mental health and dysfunctions were assessed using the German version of the GHQ-28 [24, 25]. Negatively experienced stress was assessed using the German version of the Perceived Stress Scale (PSS-10) [29]. Anxiety and depression indicators were obtained by the Patient Health Questionnaire-4 (PHQ-4 [30]). Elevated PHQ-4 scores are not used for diagnostic purposes, but instead can be understood as an indicator for possible symptoms of anxiety and depression. For all three questionnaires, the instructions were fitted to our design, asking for participants’ mental health and perceived stress in the past week.

### Assessment of stressors

Stressor load (micro-stressors), defined as chronic stressors and DHs, was conducted with the Mainz Inventory of Micro stressors [31]. The number of days a certain hassle from a list of 58 DHs occurred (ranging from 1 to 7 days) and the mean psychological strain of these events within the past 7 days were reported. In addition, we asked for exposure to COVID-19 pandemic related events (CEs) [28]. These include items such as “loss of social contacts” or “problems arranging childcare” adapted from Veer et al. [28].

### Proposed predictors

Physical distancing measures could result in increased feelings of loneliness. To test this hypothesis, the Loneliness Scale (LON) was applied [32–34]. Changes in one’s social support system during the current pandemic, which could bolster against feelings of loneliness, were assessed in week 1 using a shortened version of the Social Support Questionnaire (SOC) [35]. Positive appraisal of situations, which was proposed as a possible resilience mechanism [28], was measured by means of the Positive Appraisal Weekly Questionnaire (PAP; unpublished in-house questionnaire, see Supplementary Table 2). Coping strategies were assessed with the Brief COPE Scale [36]. In addition, using an abridged version of the Cognitive Emotion Regulation Questionnaire (CERQ) [37], especially adaptive cognitive emotion regulation strategies were measured, leaving out the non-adaptive questions of the CERQ. To assesses how participants deal with experiences and impacts of the COVID-19 pandemic, the impact of the current pandemic was measured with the COVID-19-related Positive Appraisal Questionnaire (CAP) [38]. The changes in social support during the COVID-19 pandemic were measured with the Change in Social Support Question (CSS) [28]. Further, to examine whether physical distancing recommendations were followed, a weekly Physical Distance Questionnaire was used. Questions were derived from recommendations on physical distancing from Germany’s Federal Centre for Health Education [39]. For a detailed description of the questionnaires, see Supplementary Table 3.

### Statistical analysis

Data analysis was performed using SPSS 25, R (Version 4.0.4, 2021-02-15), RStudio (Version 1.4.1106, 2009–21), and Mplus (Version 8.5). Significance was tested at 5% level. First, frequency analyses and paired samples *t*-tests were performed to ascertain the prevalence of our outcome before the lockdown and compare it with outcomes after the lockdown announcement.

The last measurement of mental health (GHQ-28), DHs, and perceived stress (PSS) prior to COVID-19-related lockdown were computed for each participant individually. The last stressor and mental health monitoring of each individual was within 3 weeks to 3 months prior to lockdown.

Subsequently, several multilevel models were analyzed to examine the relationship between mental health (assessed with the GHQ-28) and different predictors following a step-by-step procedure using the R-package nlme for linear and nonlinear mixed-effects models [40]. The hierarchical structure of the data was taken into account in all of these models. First, a null model was computed to determine the intra class coefficient (ICC), which indicates the proportion of variance that is attributable to the person. Except for SOC, which was entered grand-mean-centered, all other predictors were entered person-mean-centered, as they constitute state variables. SOC was furthermore entered on level 2, as a trait variable, whereas all other variables were entered on level 1, varying over time.

This was followed by latent class analyses with the forward modeling approach to find the optimal number of distinct trajectories, starting from a one-class to a four-class solution, considering different polynomial functions (linear, quadratic, and cubic) and within-class homogeneity vs. heterogeneity. As recommended [41], before performing a latent growth mixture model (GMM), a conventional latent growth curve model was calculated, to examine the overall fit of the data. Before taking into account the trajectory heterogeneity, a latent class growth analysis (LCGA) with fixed within-class variances (assumed within-class homogeneity), as a reduced version of the GMM (with freed intercept and fixed slope variances), was specified. Furthermore, to provide interpretive validity, the demographics age and sex were included as covariates, to predict the trajectories. The Bayesian Information Criterion (BIC) [42] as well as theoretical assumptions and plotting of the groups were used for the identification of the model that fits best with the data [43]. As recommended, 500 random sets at the initial stage and 10 final optimizations were specified to avoid local maxima [42]. Characteristics and differences between obtained classes were analyzed using analyses of variance and *χ*^2^-tests.

## Results

### Comparison of DHs and general health pre- and during lockdown

**Fig. 2:**
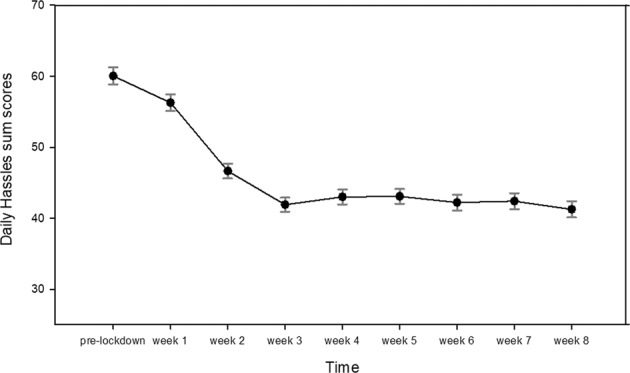
Daily hassles pre- and during lockdown. Mean scores and standard errors of the occurence of daily hassles prior to the lockdown and during weeks 1–8.

Our assessment of general micro-stressors yielded that the frequency of DHs averaged throughout the 8 weeks of assessment was significantly lower compared to the last measurement prior to the lockdown (*t*(508) = 14.61, *p* < 0.001) (Fig. 2) [26]. For detailed information on specific DHs and statistical analyses, see Supplementary Fig. 1 and Supplementary Tables 4 and 5).

**Fig. 3:**
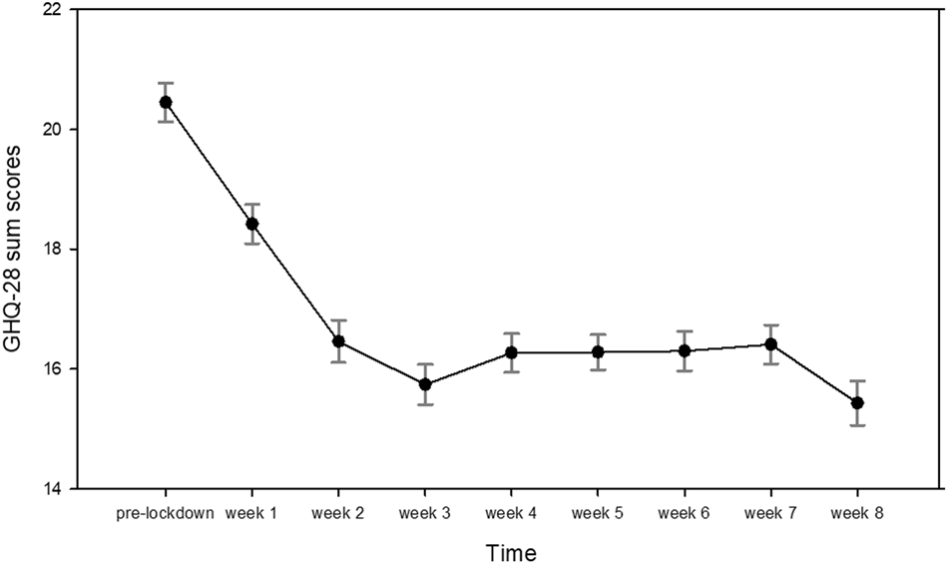
Mean scores and standard errors of mental dysfunctions (GHQ-28) prior to the lockdown and during weeks 1–8. The proposed threshold for significant distress is a total sum score of 23/24 [66].

Correspondingly, the comparison of the last measured GHQ-28 value prior to the lockdown with the averaged GHQ-28 value for the 8 weeks of lockdown assessment revealed that the mental health status significantly improved, on average, in the entire sample (*t*(508) = 8.98, *p* < 0.001) (Fig. 3) [26].

### Prediction of mental health

Perceived stress (PSS), DHs, loneliness (LON), positive appraisal specifically of the COVID-19 pandemic (CAP), critical events due to COVID-19 (CE), cognitive emotion regulation (CERQ), positive appraisal (PAP), and perceived social support (SOC) were examined as possible predictors to estimate mental health as measured by the GHQ-28 (correlations in Table 1; descriptive statistics given in Supplementary Table 6) in multilevel models.

**Table 1:** Means, standard deviations, and correlations of the predictor variables with the dependent variable (GHQ-28).

Variable	*M*	SD	GHQ	Time	PHQ	PSS	DH	LON	CAP	SOC	CE	CERQ	PAP
GHQ	16.94	8.79											
Time			−0.13**										
PHQ	1.47	1.86	0.56**	0.15**									
PSS	11.76	6.41	0.66**	−0.05**	0.68**								
DH	46.64	24.49	0.35**	−0.23**	0.17**	0.33**							
LON	8.32	2.32	−0.34**	0.02	−0.40**	−0.41**	−0.15**						
CAP	12.17	2.51	−0.26**	−0.11**	−0.29**	−0.32**	−0.02	0.23**					
SOC	30.69	4.78	−0.12**	0.00	−0.13**	−0.22**	−0.07**	0.19**	0.23**				
CE	13.05	8.37	0.10**	−0.29**	0.07**	0.11**	0.19**	−0.08**	0.00	−0.13**			
CERQ	35.31	9.36	−0.10**	−0.10**	−0.10**	−0.15**	0.09**	0.05**	0.40**	0.21**	0.02		
PAP	8.13	2.67	−0.09**	−0.14**	−0.10**	−0.13**	0.06**	0.06**	0.47**	0.16**	0.04*	0.69**	
CSS	3.13	0.64	−0.07**	−0.08**	−0.08**	−0.06**	−0.04*	0.14**	0.16**	0.13**	−0.02	0.06**	0.08**

Correlations were only calculated to determine the hierarchy of predictors entering in the model and have therefore not been corrected for multiple testing. Correlations > 0.5 are given bold face.

*CAP* positive appraisal specifically of the corona crisis, *CE* critical events due to corona, *CERQ* cognitive emotion regulation, *CSS* changes in social support, *DH* daily hassles, *GHQ* mental health (higher scores indicate worse mental health), *LON* loneliness (low values on the Loneliness Scale indicate strong feelings of loneliness), *PAP* positive appraisal, *PHQ* signs of depression and anxiety, *PSS* perceived stress, *SOC* perceived social support, *Time* measurement point.

*p < 0.05.

**p < 0.01.

First, an intercept-only model was computed to determine the influence of individual differences and the ICC (Model 1, Table 2). The results of the ICC analysis indicate that 44.63% of the variance in mental health is explained by inter-individual differences (see Supplementary Fig. 2 for every individual GHQ-28 score trajectory). Multilevel models were computed to account for that variance.

**Table 2:** Results of the multilevel models for mental health (GHQ-28).

Parameter	Model 1			Model 2			Model 3			Model 4		
Fixed effects												
	*b*	CI	*p*	*b*	CI	*p*	*b*	CI	*p*	*b*	CI	*p*
Intercept	17.06	16.51; 17.60	**<0.001**	17.01	16.46; 17.55	**<0.001**	16.98	16.43; 17.52	**<0.001**	16.63	16.04; 17.22	**<0.001**
Time linear				−70.65	−83.47; −57.83	**<0.001**	−71.33	−83.83; −54.83	**<0.001**	−8.51	−22.85; 5.82	0.245
Time quadratic				49.78	37.10; 62.45	**<0.001**	51.00	35.67; 66.33	**<0.001**	−0.94	−11.79; 9.95	0.868
PSS										0.72	0.67; 0.77	**<0.001**
DH										0.08	0.06; 0.09	**<0.001**
LON										−0.42	−0.58; −0.27	**<0.001**
CAP										−0.29	−0.42; −0.17	**<0.001**
SOC										−0.20	−0.32; −0.08	**0.002**
CE										0.01	−0.02; 0.05	0.460
CERQ										−0.07	−0.10; −0.03	**<0.001**
PAP										0.05	−0.06; 0.15	0.409
CSS										0.13	−0.28; 0.53	0.538

Random parameters												
	SD			SD			SD			SD		
Intercept	5.90	5.50; 6.34		5.90	5.50; 6.33		5.96	5.56; 6.39		6.27	5.83; 6.74	
Time linear							132.97	117.02; 151.10		79.45	61.20;103.15	
Time quadratic							116.13	99.95; 134, 93		44.57	23.21; 85.59	
Residual	6.57	6.42; 6.73		6.42	6.28; 6.57		5.75	5.59; 5.90		4.67	4.38; 4.97	
Observations	4092			4092			4092			3333		
*N*	523			523			523			491		
BIC	28,078			27,911			27,775			21,156		
*R*^2^^a^	44.63%			47.05%			57.70%			70.53%		

*BIC* Bayesian Information Criterion, *CAP* positive appraisal specifically of the COVID-19 pandemic, *CE* critical events due to COVID-19, *CERQ* cognitive emotion regulation, *CI* 95% confidence interval, *CSS* changes in social support, *DH* daily hassles, *GHQ* mental health (higher scores indicate worse mental health), *LON* loneliness (low values on the Loneliness Scale indicate strong feelings of loneliness), *PAP* positive appraisal, *PSS* perceived stress, *SOC* perceived social support, *Time* measurement point.

^a^Conditional *R*^2^.

Second, a random intercept model with time as control variable was computed (Model 2; Table 2). Time showed highly significant linear (*b* = −70.65, *p* < 0.001) and quadratic (*b* = 49.78, *p* < 0.001) influences on GHQ-28 scores. A positive quadratic influence implies that the improvement of general mental health over time (i.e., reduction of GHQ-28 score during lockdown; see above) is steeper at first but flattens over time. In the models testing the influence of the predictors, time was hence entered with a quadratic effect. Model 2 showed a conditional *R*^2^ of 47.05%.

Next, random slopes for time were entered (Model 3, Table 2). Model 3 again showed an increase in conditional *R*^2^, as 57.70% of variance could be explained and a reduction of the BIC from 27,911 in Model 2 to 27,775 in Model 3.

Last, a random slopes model with all predictors entered as fixed effects was calculated to determine the influence of the predictors on mental health (Model 4, Table 2). Mental Health (GHQ-28) served as an outcome variable and time, PSS, DH, LON, CAP, SOC, CE, CERQ, PAP, and CSS as predictors; inter-correlations are shown in Table 1.

Model 4 showed a further increase in explained variance to conditional *R*^2^ = 70.53% and further substantial reduction of the BIC to 21,156. Time as fixed effect no longer had a significant (linear or quadratic) influence. The overall decrease in GHQ-28 scores over time can thus be attributed to the influence of the other predictors. However, time as random effect was significant, as evident from the 95% confidence interval, which does not include 0—allowing the growth rate to vary randomly across individuals—indicating that the change in GHQ-28 scores over time differs significantly across individuals. Perceived stress (PSS) and DHs show a highly significant positive effect on the GHQ-28 score, i.e., increasing mental health dysfunctions (Model 4, Table 2). Decreased loneliness (LON), positive appraisal specifically of the COVID-19 pandemic (CAP), perceived social support (SOC), and increased adaptive cognitive emotion regulation (CERQ) showed significant negative influences on GHQ-28 scores, meaning less mental health problems. Critical events due to COVID-19 (CE), generally positive appraisal (PAP), and changes in social support (CSS) did not significantly predict mental health assessed by the GHQ-28.

### Latent class analysis

**Fig. 4:**
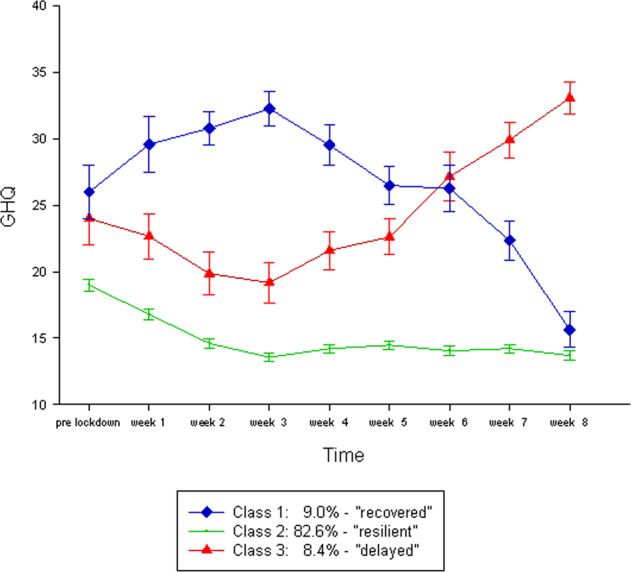
Quadratic growth mixture model (GMM) with three-class solution. Final proportions for the latent classes based on their most likely latent class membership: “recovered” class 1 blue = 9.0% (*n* = 47), “resilient” class 2 green = 82.6% (*n* = 432), “delayed dysfunction” class 3 red = 8.4% (*n* = 44).

Latent class analyses were performed to identify possible distinct subpopulations within the sample. Fit indices and a *χ*^2^-difference test indicated that a latent class growth model (LCGM) without quadratic terms did not fit the observed data as good as the model that accommodated quadratic growth. For further description, see Supplementary Methods. Subsequently, we calculated latent class growth analyses (LCGA) with fixed within-class variances, as a reduced version of latent growth mixture models (GMMs), with two through four latent classes to identify the best-fitting model. Afterwards, GMMs for the same number of classes with class-invariant intercept variances and covariances were estimated. The four-class solution has a smaller and therefore better BIC than the three-class solution. However, the smallest class contains only 27 participants, i.e., is on the verge of the recommended size of 5% of the sample [41]. Further, as it also has an inadmissible solution (i.e., negative variances), it was rejected. Moreover, the entropy value of 0.825 indicates that participants are well-assigned in the three-class model [44]. Based on these considerations and despite the disagreement of the fit indices on the optimal number of classes (Supplementary Table 7), the three-class solution was finally chosen (see Supplementary Table 8 for the growth parameters of the chosen three-class latent GMM). Moreover, sex and age as covariates were additionally included into the final class solution (for the final three-class solution with incorporated covariates, see Fig. 4; for the influence of the covariates on class membership, see Supplementary Table 9; theoretical model, see Supplementary Fig. 3).

The first of the three classes (Fig. 4, blue) contains 9.0% of the sample based on the estimated model. Notably, this class has a mean GHQ-28 value beyond the threshold of 23/24 points [24] before the lockdown (*M*_pre-lockdown_ = 25.72) and deteriorates significantly in its mental health until week 3 (*M*_week3_ = 32.26). From week 4 onwards, mental health improves steadily until the mean value is considerably below the GHQ-28 threshold in week 8 (*M*_week8_ = 15.64). Due to its trajectory in GHQ-28 scores, this class was labeled “recovered.” The second and largest class (Fig. 4, green) comprises 82.6% of the participants and starts below the GHQ-28 threshold with a mean of *M*_pre-lockdown_ = 19.48 prior to lockdown announcements and decreases with a small plateau in week 4 to an average value of *M*_week8_ = 13.69. It was therefore labeled as “resilient.” Class three (Fig. 4, red) covers 8.4% of the participants and also has an initial GHQ-28 value over 23/24 of *M*_pre-lockdown_ = 24.26 prior to lockdown announcements, declines continuously until week 3 to an average of *M*_week3_ = 19.17 but then increases steadily to *M*_week8_ = 33.06 in week 8. It was therefore termed “delayed dysfunction” (see also ref. [26]). Labels were chosen analogous to those used in related work on mental health trajectories during adversity [45]. Of note, measurements indicated as “pre-lockdown” constitute the last measurement before the lockdown for each individual. However, these values are not identical with measures at the individual baseline of participants when entering the LORA study. Noteworthy, participants in class two reported significantly less DHs (*M* = 57.28, SD = 25.69) prior to the lockdown compared to subjects in the other two classes (*M*_class1_ = 73.13, SD = 28.74, *M*_class3_ = 72.98, SD = 32.33, *F*(2,506) = 12.94, *p* < 0.001). Class 1 is significantly younger (*F*(2,520) = 8.20, *p* < 0.001), entails more women (*χ*^2^(2) = 10.63, *p* = 0.005), and less participants in this class have children under the age of 18 years living in their household (*χ*^2^(2) = 6.77, *p* = 0.034) compared to the other two classes. For socio-demographic details and a comparison between the resulting classes, see Table 3.

**Table 3:** Class demographics.

	Class 1 “recovered” (*n* = 47, 9%)	Class 2 “resilient” (*n* = 432, 82.6%)	Class 3 “delayed dysfunction” (*n* = 44, 8.4%)	Test statistic	*p*-Value
Gender (m/f)	5/42	146/286	13/31	*χ*^2^(2) = 10.63	0.005^**^
In percent	10.6%/89.4%	33.8%/66.2%	29.5%/70.5%		
Age, *M* (SD)	26.98 (5.02)	31.88 (8.51)	33.05 (8.82)	*F*(2520) = 8.20	<0.001^**^
Relationship status, *n* (%)				*χ*^2^(4) = 2.50	0.644
In a relationship	29 (65.9%)	285 (67.4%)	30 (68.2%)		
Not in a relationship	14 (31.8%)	136 (32.2%)	14 (31.8%)		
Other	1 (2.3%)	2 (0.5%)	0 (0%)		
Children < 18 yrs in household, true for *n* (%)	3 (7.0%)	102 (24.3%)	11 (25.0%)	*χ*^2^(2) = 6.77	0.034*
Employment status, *n* (%^a^)					
Full-time	17 (36.2%)	177 (41.0%)	20 (45.5%)	*χ*^2^(2) = 0.81	0.666
Part-time	6 (12.8%)	91 (21.1%)	9 (20.5%)	*χ*^2^(2) = 1.81	0.405
Obtaining a degree	21 (44.7%)	142 (32.9%)	19 (43.2%)	*χ*^2^(2) = 4.09	0.129
Unemployed	2 (4.3%)	10 (2.3%)	1 (2.3%)	*χ*^2^(2) = 0.668	0.716
Other	4 (8.5%)	56 (13.0%)	2 (4.5%)	*χ*^2^(2) = 3.26	0.196

Percentages indicate percent within a class.

*f* female, *m* male, *yrs* years.

^a^Several options could be chosen per participant.

**Fig. 5:**
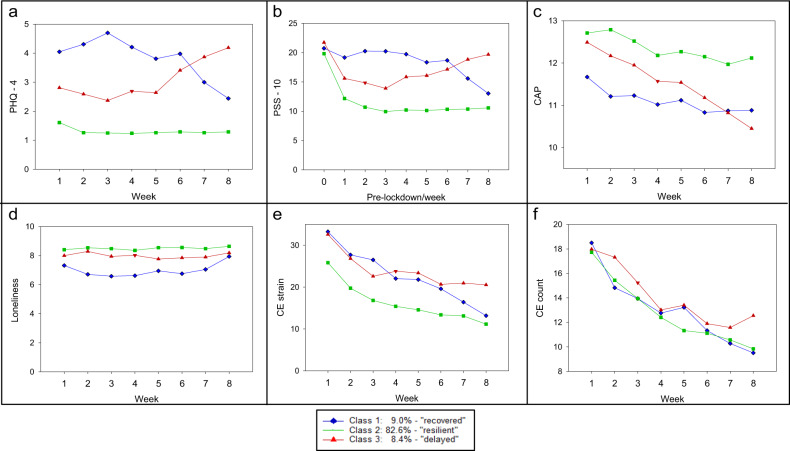
Comparison of three latent classes (see Fig. 4) over the 8-week time frame. **a** PHQ-4 means, **b** PSS-10 means, **c** positive appraisal sum scores (CAP), **d** mean loneliness scores (low values on the Loneliness Scale indicate strong feelings of loneliness), **e** mean of COVID-19-related events strain, **f** mean frequency of COVID-19-related events. PHQ-4, mean of signs of depression and anxiety; PSS-10, perceived stress; Class 1 blue, “recovered”; Class 2 green, “resilient”; Class 3 red, “delayed dysfunction”.

In addition, the three classes (based on the participant’s most likely latent class membership) were compared in detail over the 8 weeks of assessment with respect to possible COVID-19-related factors influencing mental health outcomes. For validation of the GHQ-28 scores, depression and anxiety symptoms assessed with the PHQ-4 are additionally reported. For the trajectories of these factors between the groups, see Fig. 5 and the Supplement for a detailed description.

## Discussion

In this study we explored the temporal dynamics of the lockdown measures with regard to perceived stress and strains, as well as mental health outcomes. Experienced micro-stressors were reduced, which contradicts reports indicating general negative mental health consequences due to the pandemic [19, 46–49]. This is also in contrast to data from countries that underwent a complete lockdown, such as China [48] and Italy [5], reporting an increase in mental health problems [2, 50]. Compared to those countries, governmental control measures of quarantine were relatively lenient in Germany, with no full lockdown [51]. Further, various political and economic instruments, including a massive economic support program, buffered the detrimental economic consequences of the pandemic in Germany, so that incidence rates of reporting (fear of) job loss or financial losses were a third lower in Germany than in other European countries [28]. As a possible result of the different restrictions and direct economic effects, the majority of German studies, including the present one, did not find a general decrease in mental health during the first German lockdown. Even more, we found a general increase in mental health, indicating that mental health functioning can also improve after exposure to adverse events, a phenomenon called psychosocial gain from adversity. This effect is different from posttraumatic growth, as it does not require internal psychological processes, but results from changes in the social environment due to a jointly experienced adversity [52], such as the current COVID-19 pandemic.

As opposed to uniform and general effects of lockdown on mental health, we identified demographic and psychological predictors for mental health outcomes. We were able to predict pronounced mental health dysfunctions with stress indicators, such as the number of DHs, subjectively perceived stress, and feeling lonelier. Responsible for improved mental health were positive appraisal specifically of the COVID-19 pandemic, perceived social support, and increased adaptive cognitive emotion regulation. These findings are in line with a study investigating exposure to the European COVID-19 pandemic lockdown by Veer et al. [28]. This study also identified positive appraisal style specifically of the COVID-19 pandemic as the most important factor for coping with the current circumstances, which is very similar to our concept of COVID-19-specific positive appraisal [28]. Notably, the usefulness of specific positive appraisal of the dominant stressor seems to exceed general positive appraisal, the latter being no significant predictor for mental health outcomes in the present study. The finding that social support in general is predictive of mental health outcomes is also in line with previous studies [28, 53]. However, we found no change in social support during the COVID-19 pandemic. Consequentially, the variable was not a suitable predictor for mental health. Adaptive cognitive emotion regulation, on the other hand, was a significant predictor, which is not surprising, as it has been linked to positive long-term mental health outcomes [54, 55]. Interestingly, the frequency of critical events due to COVID-19 did not predict mental health. A possible explanation may be that chronic stressors and DHs play a greater role than the (comparatively rare) critical events due to COVID-19, especially after accustoming to the circumstances.

Even though we were able to observe a slight improvement of overall mental health outcomes in our sample, adjusting to the new circumstances may well have deleterious effects on vulnerable groups, such as people with or at risk for depression or anxiety disorders [56–58]. Therefore, we investigated the possible existence of different subgroups among our sample. Accordingly, we identified three subclasses with distinct mental health: a “resilient” group with initially improved mental health over time, a “recovered” group with an initially high mental dysfunction increase until week 3, followed by a quick return to baseline, and a “delayed dysfunction” group that showed a significant deterioration of mental health after week 4 of the assessment period. Notably, there were no major demographic differences between the groups and they did not differ in cohabitation with a partner. Likewise, a study by Hawryluck et al. [59] on the consequences of quarantine after potential SARS exposure found that marital status is an irrelevant predictor for mental health status. Notably though, there was a significant difference in the amount of participants living with children under the age of 18 years in their household. The recovered group entailed significantly less participants living with minors than the other two groups. However, this difference is most likely due to the significantly younger mean age in this group compared to the other two groups.

The delayed dysfunction and recovered groups found in the present study are of particular interest, as they possibly entail susceptible people, who may need special support during a pandemic to prevent deleterious mental health outcomes. Apart from the commonality of poor mental health values before the COVID-19 pandemic in both groups, they showed distinct trajectories for perceived stress, COVID-19-specific positive appraisal, and straining from COVID-19-related events. Regarding perceived stress, the recovered group showed a reduction in initially high stress levels and loneliness after COVID-19 regulations started to be relaxed, while the vulnerable group displayed an exacerbation of stress levels at the same time. In addition, although the number of subjectively reported COVID-19-specific events did not differ between all groups, data from the delayed dysfunction group suggests that as mental health deteriorated, COVID-19-specific events were experienced as more straining and vice versa. This is accompanied by a reduction in the ability to appraise these events. The resilient group, on the other hand, was constantly able to positively appraise the COVID-19-specific events and shows a reduction of straining due to them over time. This positive appraisal might have helped this group to bolster the straining influence of COVID-19-specific events. Likewise, the recovered participants show a stable appraisal tendency after an initial decrease until week 4, which is accompanied by less experienced strain. Hence, the recovered group struggles at first upon the introduced lockdown measures but seems to adapt over time. This is in accordance with the assumptions made by the psychosocial gain of adversity model, assuming the highest gain for those subjects, who are susceptible for psychological malfunction [60].

Interestingly, there was no change in perceived loneliness in the delayed dysfunction group and only a slight change in the recovered group. As mentioned earlier, the SOEP-CoV study reached a similar conclusion. Although that study found that people in Germany were lonelier than in previous years, symptoms of depression and anxiety did not increase [8]. Therefore, loneliness in times of a pandemic does not necessarily or immediately lead to poorer mental health. The increase in loneliness is likely perceived as collective and can be attributed to external circumstances. The same may hold true for perceived social support and emotion regulation, for which we also found no significant differences in the trajectories between the three latent classes.

### Limitations

Some limitations of the present study must be considered. There might have been a selection bias, as only a subsample from the LORA study was willing to participate. Notably though, participants in the current study were representative of the full LORA sample in terms of socio-demographic aspects. Nonetheless, these participants might have differed from the total LORA sample in the amount of experienced micro-stressors, their stressor load, or other factors during the lockdown not evident from the data at hand. Still, we found a relevant variance in exposure to micro-stressors within our sample, which increases the generalizability of the findings. It must be pointed out, although, that also the full LORA sample is not fully representative of the general population, as it entails an overrepresentation of younger and well-educated participants. Moreover, the investigated period of 8 weeks only covers the full time of the lockdown measures and about 2 weeks after restrictions had been relaxed. However, certain effects of the COVID-19 pandemic might only affect mental health after a longer period due to chronic wearing. Still, we were able to observe a deterioration of mental health in vulnerable participants, also for this short time interval. Hence, the governmental measures obviously had an immediate effect on these participants. Furthermore, as was mentioned earlier, the restrictions in Germany were comparably mild, as only the number of contact persons was limited, which could have had a positive impact on participants’ mental health.

### Relevance and recommendations

The present study is among the first to longitudinally investigate the impact of the COVID-19 pandemic related lockdown measures applying both a high sampling rate and investigating a pre-existing sample. This sample was deep-phenotyped prior to the COVID-19 pandemic, which allows for a benchmark comparison. This enabled the identification of heterogeneous mental health trajectories through latent class analyses, thereby adding important information to the finding that mental health can also improve during lockdown under certain conditions. The identification of vulnerable subjects in times of adversity is of utmost importance, to prevent the manifestation of mental disorders. Furthermore, interventions to alleviate psychological impacts of lockdown on mental health should specifically address participants at risk for developing a mental disorder, i.e., vulnerable participants with already reduced general mental health prior to the pandemic, rather than large-scale interventions for the entire population. It is up to future studies to develop tailored interventions for these vulnerable subjects. The group of recovered participants maintained mentally healthy and likely applied the identified mechanisms predicting mental health, such as cognitive emotion regulation.

Therefore, a first starting point for practical interventions could be the identified possible protective factors and mechanisms, i.e., positive appraisal of the COVID-19 pandemic and emotion regulation, for stable mental health in the present study. Implementing these mechanisms may be beneficial to remain mentally healthy, as in the resilient group, or to bounce back to baseline levels, as observed in the recovered group. Much remains to be learned about the factors that influence who can and who cannot maintain mental health in the face of large-scale crises. Especially the further investigation of different factors and mechanisms working on groups with an increased risk of becoming mentally ill is of particular relevance for the future.

## Supplementary information

Supplemental Material

## References

[1] Röhr S, Müller F, Jung F, Apfelbacher C, Seidler A, Riedel-Heller SG (2020). Psychosocial impact of quarantine measures during serious coronavirus outbreaks: a rapid review. Psychiatr Prax.

[2] Brooks SK, Webster RK, Smith LE, Woodland L, Wessely S, Greenberg N (2020). The psychological impact of quarantine and how to reduce it: rapid review of the evidence. Lancet.

[3] Wang C, Pan R, Wan X, Tan Y, Xu L, Ho CS, et al. Immediate psychological responses and associated factors during the initial stage of the 2019 coronavirus disease (COVID-19) epidemic among the general population in China. Int J Environ Res Public Health. 2020;17:1729. 10.3390/ijerph17051729 PMC7084952 32155789

[4] Xiong J, Lipsitz O, Nasri F, Lui L, Gill H, Phan L (2020). Impact of COVID-19 pandemic on mental health in the general population: a systematic review. J Affect Disord.

[5] Rossi R, Socci V, Talevi D, Mensi S, Niolu C, Pacitti F, et al. COVID-19 pandemic and lockdown measures impact on mental health among the general population in Italy. An N=18147 web-based survey. medRxiv:2020.04.09.20057802 [Preprint]. 2020. Available from: 10.1101/2020.04.09.20057802. 10.3389/fpsyt.2020.00790 PMC7426501 32848952

[6] Vindegaard N, Benros ME (2020). COVID-19 pandemic and mental health consequences: systematic review of the current evidence. Brain Behav. Immun.

[7] Pan K-Y, Kok AAL, Eikelenboom M, Horsfall M, Jörg F, Luteijn RA, et al. The mental health impact of the COVID-19 pandemic on people with and without depressive, anxiety, or obsessive-compulsive disorders: a longitudinal study of three Dutch case-control cohorts. Lancet Psychiatry. 2020;8:121–9. 10.1016/S2215-0366(20)30491-0 PMC7831806 33306975

[8] Entringer, T., Kröger, H. Einsam, aber resilient – Die Menschen haben den Lockdown besser verkraftet als vermutet. DIW Aktuell. 2020;46:6S.

[9] Schäfer SK, Sopp MR, Schanz CG, Staginnus M, Göritz AS, Michael T (2020). Impact of COVID-19 on public mental health and the buffering effect of a sense of coherence. Psychother Psychosom..

[10] Lai J, Ma S, Wang Y, Cai Z, Hu J, Wei N (2020). Factors associated with mental health outcomes among health care workers exposed to coronavirus disease 2019. JAMA Netw Open.

[11] Zhang WR, Wang K, Yin L, Zhao WF, Xue Q, Peng M (2020). Mental health and psychosocial problems of medical health workers during the COVID-19 epidemic in China. Psychother Psychosom.

[12] Li LZ, Wang S (2020). Prevalence and predictors of general psychiatric disorders and loneliness during COVID-19 in the United Kingdom. Psychiatry Res.

[13] Pieh C, Budimir S, Probst T. The effect of age, gender, income, work, and physical activity on mental health during coronavirus disease (COVID-19) lockdown in Austria. J Psychosom Res. 2020;136:110186. 10.1016/j.jpsychores.2020.110186 PMC7832650 32682159

[14] Horesh D, Kapel Lev-Ari R, Hasson-Ohayon I (2020). Risk factors for psychological distress during the COVID-19 pandemic in Israel: loneliness, age, gender, and health status play an important role. Br J Health Psychol.

[15] Liang L, Ren H, Cao R, Hu Y, Qin Z, Li C, et al. The Effect of COVID-19 on Youth Mental Health. Psychiatr Q. 2020:1–12. 10.1007/s11126-020-09744-3. 10.1007/s11126-020-09744-3 PMC7173777 32319041

[16] Ni MY, Yang L, Leung C, Li N, Yao XI, Wang Y (2020). Mental health, risk factors, and social media use during the COVID-19 epidemic and cordon sanitaire among the community and health professionals in Wuhan, China: cross-sectional survey. JMIR Ment Health..

[17] Zhou Y, Macgeorge EL, Myrick JG (2020). Mental health and its predictors during the early months of the covid-19 pandemic experience in the United States. Int J Environ Res Public Health.

[18] Agha S (2021). Mental well-being and association of the four factors coping structure model: a perspective of people living in lockdown during COVID-19. Ethics Med Public Health.

[19] Pierce M, Hope H, Ford T, Hatch S, Hotopf M, John A, et al. Mental health before and during the COVID-19 pandemic: a longitudinal probability sample survey of the UK population. Lancet Psychiatry. 2020;7:P883–92. 10.1016/S2215-0366(20)30308-4. 10.1016/S2215-0366(20)30308-4 PMC7373389 32707037

[20] Sønderskov KM, Dinesen PT, Santini ZI, Østergaard SD. The depressive state of Denmark during the COVID-19 pandemic. Acta Neuropsychiatr. 2020;32:226–8. 10.1017/neu.2020.15. 10.1017/neu.2020.15 PMC7176490 32319879

[21] Fancourt D, Steptoe A, Bu F. Trajectories of anxiety and depressive symptoms during enforced isolation due to COVID-19 in England: a longitudinal observational study. Lancet Psychiatry. 2021;8:P1421–9. 10.1016/S2215-0366(20)30482-X PMC7820109 33308420

[22] Mancini AD (2020). Heterogeneous mental health consequences of COVID-19: costs and benefits. Psychol Trauma.

[23] Chmitorz A, Neumann RJ, Kollmann B, Ahrens KF, Öhlschläger S, Goldbach N, et al. Longitudinal determination of resilience in humans to identify mechanisms of resilience to modern-life stressors: the longitudinal resilience assessment (LORA) study. Eur Arch Psychiatry Clin Neurosci. 2020. 10.1007/s00406-020-01159-2. 10.1007/s00406-020-01159-2 PMC8354914 32683526

[24] Goldberg, D. Manual of the general health questionnaire. Windsor: NFER Publishing Company; 1978.

[25] Klaiberg A, Schumacher J, Brähler E (2004). General Health Questionnaire 28 - Statistical testing of a German version with a representative sample of the general population. Z Klin Psychol Psychiatr Psychother.

[26] Ahrens KF, Neumann RJ, Kollmann B, Plichta MM, Lieb K, Tüscher O, et al. Differential impact of COVID-related lockdown on mental health in Germany. World Psychiatry. 2021;20:140–1. 10.1002/wps.20830 PMC7801843 33432755

[27] Kalisch R, Müller MB, Tüscher O. A conceptual framework for the neurobiological study of resilience. Behav Brain Sci. 2015;38:e92. 10.1017/S0140525X1400082X. 10.1017/S0140525X1400082X 25158686

[28] Veer IM, Riepenhausen A, Zerban M, Wackerhagen C, Puhlmann LMC, Engen H, et al. Mental resilience in the Corona lockdown: first empirical insights from Europe. PsyArXiv Prepr. 2020. 10.31234/osf.io/4z62t.

[29] Cohen S, Kamarck T, Mermelstein R (1983). A global measure of perceived stress. J Health Soc Behav.

[30] Löwe B, Wahl I, Rose M, Spitzer C, Glaesmer H, Wingenfeld K (2010). A 4-item measure of depression and anxiety: validation and standardization of the Patient Health Questionnaire-4 (PHQ-4) in the general population. J Affect Disord.

[31] Chmitorz A, Kurth K, Mey LK, Wenzel M, Lieb K, Tüscher O (2020). Assessment of microstressors in adults: questionnaire development and ecological validation of the Mainz inventory of microstressors. JMIR Ment Heal.

[32] Hughes ME, Waite LJ, Hawkley LC, Cacioppo JT (2004). A short scale for measuring loneliness in large surveys results from two population-based studies. Res Aging.

[33] Hawkley LC, Duvoisin R, Ackva J, Murdoch JC, Luhmann, M. Loneliness in Older Adults in the USA and Germany: Measurement Invariance and Validation. Working Paper Series, NORC at the University of Chicago; 2015.

[34] Richter D, Schupp J. SOEP innovation sample (SOEP-IS) — description, structure and documentation. SSRN Electron J. 2012. 10.2139/ssrn.2131214.

[35] Fydrich T, Geyer M, Hessel A, Sommer G, Brähler E. Fragebogen zur Sozialen Unterstützung (F-SozU): Normierung an einer repräsentativen Stichprobe. Diagnostica. 1999;45:212–6.

[36] Carver CS (1997). You want to measure coping but your protocol’s too long: consider the brief COPE. Int J Behav Med.

[37] Loch N, Hiller W, Witthöft M (2011). Der Cognitive Emotion Regulation Questionnaire (CERQ): Erste teststatistische Überprüfung einer deutschen Adaption. Z Klin Psychol Psychother.

[38] COSMO. Fragebogen Welle 4 24.03.−25.03.2020. 2020. Available at: https://dfncloud.uni-erfurt.de/s/Cmzfw8fPRAgzEpA#pdfviewer.

[39] Bundeszentrale für gesundheitliche Aufklärung. Verhaltensregeln und -empfehlungen zum Schutz vor dem Coronavirus SARS-CoV-2 im Alltag und im Miteinander. 2020. https://www.bzga.de/presse/pressemitteilungen/2020-08-24-bzga-ruft-auf-die-aha-formel-gewissenhaft-einzuhalten/. Accessed 6 May.

[40] Pinheiro J, Bates D, DebRoy S, Sarkar D, R Core Team. nlme: Linear and Nonlinear Mixed Effects Models. R package version 3.1-152. 2020. https://CRAN.R-project.org/package=nlme.

[41] Wickrama KAS, Lee T, O’Neal CW, Lorenz FO. Higher-Order Growth Curves and Mixture Modeling with Mplus: A Practical Guide. 1st edn. New York: Taylor & Francis Group, Routledge; 2016.

[42] Nylund KL, Asparouhov T, Muthén BO (2007). Deciding on the number of classes in latent class analysis and growth mixture modeling: a Monte Carlo simulation study. Struct Equ Model A Multidiscip J.

[43] van de Schoot R, Sijbrandij M, Winter SD, Depaoli S, Vermunt JK (2017). The GRoLTS-checklist: guidelines for reporting on latent trajectory. Stud Struct Equ Model.

[44] Celeux G, Soromenho G (1996). An entropy criterion for assessing the number of clusters in a mixture model. J Classif.

[45] Bonanno GA, Westphal M, Mancini AD (2011). Resilience to loss and potential trauma. Annu Rev Clin Psychol.

[46] Kumar A, Nayar KR (2020). COVID 19 and its mental health consequences. J Ment Health.

[47] World Health Organization. Mental health and psychosocial considerations during COVID-19 outbreak. Frankfurt am Main, Germany: World Health Organization; 2020. https://www.who.int/publications/i/item/WHO-2019-nCoV-MentalHealth-2020.1. Accessed 6 May 2020.

[48] Wang C, Pan R, Wan X, Tan Y, Xu L, McIntyre RS (2020). Brain, behavior, and immunity. A longitudinal study on the mental health of general population during the COVID-19 epidemic in China. Brain Behav Immun.

[49] Li W, Yang Y, Liu ZH, Zhao YJ, Zhang Q, Zhang L (2020). Progression of mental health services during the COVID-19 outbreak in China. Int J Biol Sci.

[50] Rajkumar RP (2020). COVID-19 and mental health: a review of the existing literature. Asian J Psychiatr.

[51] Bundesregierung. 6. Mai 2020: Regeln zum Corona-Virus. (Bundesregierung; Frankfurt am Main, Germany, 2020). https://www.bundesregierung.de/breg-de/leichte-sprache/6-mai-2020-regelnzum-corona-virus-1755252. Accessed 6 May 2020.

[52] Mancini AD (2019). When acute adversity improves psychological health: a social-contextual framework. Psychol. Rev..

[53] Zhou S-J, Zhang L-G, Wang L-L, Guo Z-C, Wang J-Q, Chen J-C, et al. Prevalence and socio-demographic correlates of psychological health problems in Chinese adolescents during the outbreak of COVID-19. Eur Child Adolesc Psychiatry. 2020. 10.1007/s00787-020-01541-4. 10.1007/s00787-020-01541-4 PMC7196181 32363492

[54] Garnefski N, Kraaij V (2007). The cognitive emotion regulation questionnaire psychometric features and prospective relationships with depression and anxiety in adults. Eur J Psychol Assess.

[55] Kalisch R, Baker DG, Basten U, Boks MP, Bonanno GA, Brummelman E (2017). The resilience framework as a strategy to combat stress-related disorders. Nat Hum Behav.

[56] Sprang G, Silman M (2013). Posttraumatic stress disorder in parents and youth after health-related disasters. Disaster Med Public Health Prep.

[57] Zhu Y, Chen L, Ji H, Xi M, Fang Y, Li Y (2020). The risk and prevention of novel coronavirus pneumonia infections among inpatients in psychiatric hospitals. Neurosci Bull.

[58] Yao H, Chen J-H, Xu Y-F. Patients with mental health disorders in the COVID-19 epidemic. Lancet Psychiatry. 2020;7:e21. 10.1016/S2215-0366(20)30090-0 PMC7269717 32199510

[59] Hawryluck L, Gold WL, Robinson S, Pogorski S, Galea S, Styra R (2004). SARS control and psychological effects of quarantine, Toronto, Canada. Emerg Infect Dis.

[60] Mancini AD (2019). When acute adversity improves psychological health: a social-contextual framework. Psychol Rev.

[61] Robert Koch Institut. Täglicher Lagebericht des RKI zur Coronavirus-Krankheit-2019 (COVID-19) 09.03.2020 –AKTUALISIERTER STAND FÜR DEUTSCHLAND KORRIGIERTE VERSION (10.03.2020). 2020.

[62] World Health Organization. WHO Health Emergency Dashboard. 2020. Available at: https://covid19.who.int/region/euro/country/de. Accessed 30 July 2020.

[63] Bundesministerium des Innern, für B. und H. Vorübergehende Grenzkontrollen an den Binnengrenzen zu Österreich, der Schweiz, Frankreich, Luxemburg und Dänemark. 2020.

[64] Bundesregierung. Besprechung der Bundeskanzlerin mit den Regierungschefinnen und Regierungschefs der Länder - Die Bundeskanzlerin und die Regierungschefinnen und Regierungschefs der Länder fassen am 22. März 2020 folgenden Beschluss. 2020. Available at: https://www.bundesregierung.de/breg-de/themen/coronavirus/besprechung-der-bundeskanzlerin-mit-den-regierungschefinnen-und-regierungschefs-der-laender-1733248.

[65] Tagesschau. Maskenpflicht in allen Bundesländern. (2020). https://www.tagesschau.de/inland/coronamaskenpflicht-103.html, accessed on May 11th 2020.

[66] Goldberg DP, Hillier VF (1979). A scaled version of the General Health Questionnaire. Psychol. Med..

