# Supplementary material for: Impact of COVID-19 lockdown on mental health in Germany: longitudinal observation of different mental health trajectories and protective factors
Source: Transl Psychiatry. 2021 Jul 17;11:392. doi: 10.1038/s41398-021-01508-2 (PMC8287278; doi:10.1038/s41398-021-01508-2)
Supplement: Supplementary file 1 — Supplemental Material [file 41398_2021_1508_MOESM1_ESM.docx]

**Supplemental Methods**

*Sample distribution*

Participants’ media usage regarding with the influence of consequences of the pandemic on the economy, climate, etc. and even more so, their occupation with the number of infections according case numbers gradually decreased from week 1-8, from *n* = 433 participants checking infection numbers rates in week 1 to *n* = 230 in week 8 and *n* = 438 participants engaging themselves with the pandemic’s influence on the economy and climate in week 1 to *n* = 273 participants in week 8. While they gathered most information on the number of infections from television and the Robert Koch Institute, television, newspapers, and social media were the most frequented sources for information about the influence of the pandemic on the economy, climate, etc. The source type selected remained stable throughout the eight weeks of assessment (see also Supplementary Table 1 for a demographic overview).

*Latent class analyses*

The latent class growth model (LCGM) indicated the existence of subpopulations with distinct trajectories due to statistically significant variances and relatively poor fit indices (CFI = .87, TLI = .88, RMSEA = .11, SRMR =.13)^41^.

For the LGCM the intercept (17.87, *p* <.001) and the slope (-0.30, *p* < .001) were statistically significant. The mean trajectory of mental dysfunction (GHQ-28) declined over time. The variances of the intercept (47.27, *p* < .001) and slope (0.69, *p* < .001) were also significant. This indicates that the individuals differ in their initial levels of mental health and change in mental health over the 8 weeks.

Moreover, there was a significant negative covariance between the intercept and slope (-2.60, *p* < .001), which indicates, that on average participants with high initial levels of dysfunctions were more likely to experience improvement of their mental health over time.

Next, a quadratic LGCM was estimated to test for non-linear changes over time in mental health. Compared to the linear LGCM the overall model fit indices of the quadratic model showed improvement (CFI = .92, TLI = .92, RMSEA = .09, SRMR =.08) and the average intercept was slightly higher (19.30, *p* < .001). The linear slope still decreased over time (-1.22, *p* < .001), and the quadratic trajectory increased significantly over time (0.11, *p* < .001) and pointed to an accelerating rate of change. The significant variances of the intercept, linear, and quadratic trajectories indicated inter-individual differences (31.166 *p* < .001; 3.271, *p* = .001; 0.06, *p* < .001, respectively). Covariances among the intercept, linear slope, and the intercept and quadratic slope were not statistically significant, but the covariances between the quadratic and the linear slope were statistically significant (-0.41, p < .001).

The nested χ² difference test was used to compare the linear and quadratic models. The χ² of the linear model is 285.733 and df = 40. The χ² of the quadratic model is 182.014 and df = 36. χ²_DIFF_ (4) = 103.719. Based on an alpha = .05 the critical value for χ²_DIFF_ = 9.487 the quadratic model seems to have a significantly better fit to the data.

Furthermore, a cubic LGCM was estimated. Compared to the quadratic LGCM the model fit indices did not improve significantly (CFI = .92, TLI = .92, RMSEA = .09, SRMR= .13), and the BIC of the cubic LGCM showed no improvement (BIC_quadratic_ = 27586.13, BIC_cubic_ = 27603.61), therefore the cubic model was discarded.

*Description of relevant factor trajectories in subgroups*

The trajectory of PHQ-4 scores mimicked that observed for the GHQ-28 score, with *resilient* scores in the *resilient* class throughout the weeks, a sharp decrease in scores around week 6 in class 1, and a pronounced increase in scores at the same time in the *delayed dysfunction* class (Figure 5a).

Regarding perceived stress (Figure 5b), PSS scores were roughly the same in all three classes prior to the lockdown. Participants in the *recovered* class displayed the highest scores (*M* = 19.17, *SD* = 6.77), which showed a steep decline from week 6 (*M* = 18.68, *SD* = 6.67) until week 8 (*M* = 13.06, *SD* = 5.51), indicating a pronounced and significant reduction in perceived social stress (paired *t*-test: *t*(31) = 5.11, *p* < .001). Subjects in the *resilient* class reported the least amount of perceived stress in week 1 (*M* = 12.17, *SD* = 6.26) and their stress level decreased significantly further until it reached a plateau in week 3 (*M* = 9.94, *SD* = 5.18; paired *t*-test: *t*(353) = 8.37, *p* < .001). As such, these participants seemed to be only mildly stressed by the lockdown regulations. Subjects in the *delayed dysfunction* class had a sharp increase in their perceived stress level from *M* = 13.90 (*SD* = 7.90) in week 3 to *M* = 19.68 (*SD* = 6.82) in week 8 (paired *t*-test: *t*(29) = -4.50, *p* < .001).

Considering the positive appraisal specifically related to the pandemic situation (Figure 5c), participants in the *resilient* and *delayed dysfunction* classes started off at roughly the same mean values in week 1 (*resilient* class: *M* = 12.71, *SD* = 2.44; *delayed dysfunction* class: *M* = 12.49, *SD* = 2.58; *t*(432) = -.54, *p* = .59). While participants in the *resilient* class were constantly able to positively appraise the situation, participants in the *delayed dysfunction* class showed a steady decrease in positive appraisal scores until week 8 (*M* = 10.45, *SD* = 3.38; week 1 vs. week 8: *t*(28) = 3.31, *p* = .003). Subjects in the *recovered* class had the lowest positive appraisal scores in week 1 (*M* = 11.67, *SD* = 2.76), which remained low throughout the assessment (week 8: *M* = 10.88, *SD* = 2.11).

The loneliness scores for the three classes (Figure 5d) were quite similar in week 1 (*recovered* class: *M* = 7.32, *SD* = 2.93; *resilient* class: *M* = 8.41, *SD* = 2.22; *delayed dysfunction* class: *M* = 7.32, *SD* = 2.93; only the comparison between the *recovered* and the *resilient* classes became significant, *F*(2,472) = 4.54, *p* = .011, post-hoc comparison: p = .004), but followed different trajectories thereafter. While participants in the *resilient* class continued to show little loneliness, subjects in the *recovered* class had consistently lower scores (i.e., higher loneliness) than the other two classes, which steadily increased again from week 6 onwards (week 6: *M* = 6.76, *SD* = 2.56; week 8: *M* = 7.94, *SD* = 2.40), indicating a reduction in loneliness after the lockdown regulations were relaxed.

Participants in the *resilient* class showed an initially quite low straining score from pandemic-related events (*M* = 25.85, *SD* = 13.90; *F*(2,473) = 8.20, *p* < .001), which continuously decreased thereafter until week 8 (*M* = 11.15, *SD* = 10.03; week 1 vs. 8: *t*(298) = 22.79*, p* < .001). In contrast, straining from corona related events initially decreased in the *recovered* and *delayed dysfunction* classes as well (week 1: *recovered* class *M* = 33.26, *SD* = 17.07, *delayed dysfunction* class *M* = 32.59, *SD* = 17.00), but remained higher in the *delayed dysfunction* class than in the *recovered* class (*recovered* class week 8: *M* =13.17, *SD* = 8.51; *delayed dysfunction* class week 8: *M* = 20.55, *SD* = 19.45), matching the trajectory of PSS scores in the *delayed dysfunction* and *recovered* class in weeks 6-8. However, this difference did not reach significance (*t*(40.02) = 1.95, *p* = .06). Notably, the amount of corona related events was not significantly different between the three classes throughout the assessment (all *p* > .05). From this finding, a subjective experience of stress and straining of events may be inferred (Figure 5e, f).

**Supplemental Figures**

**
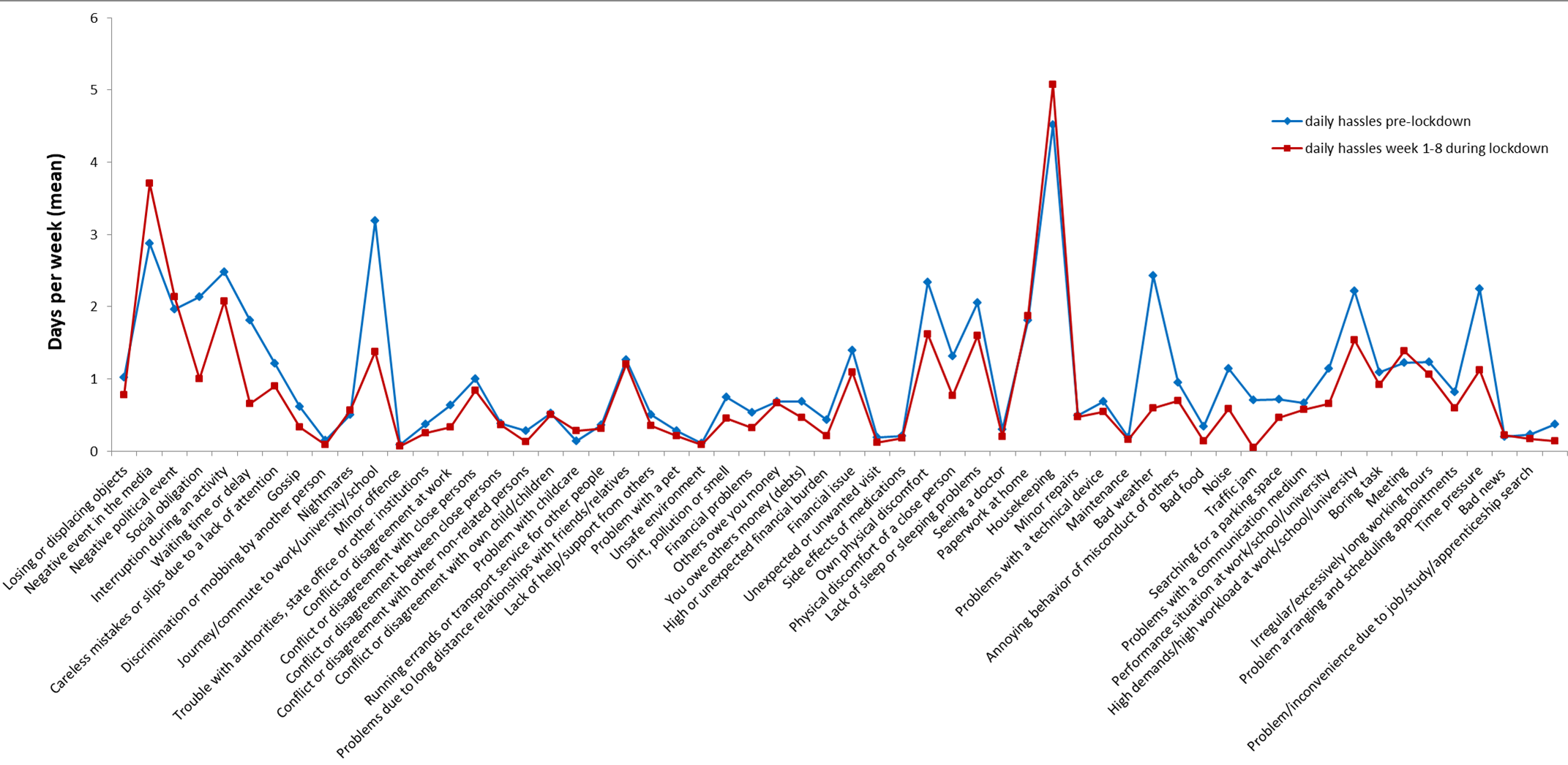
**

Supplementary Fig.1. Daily hassles items mean frequency per week: contrast between daily hassles pre-lockdown and week 1-8 during lockdown.


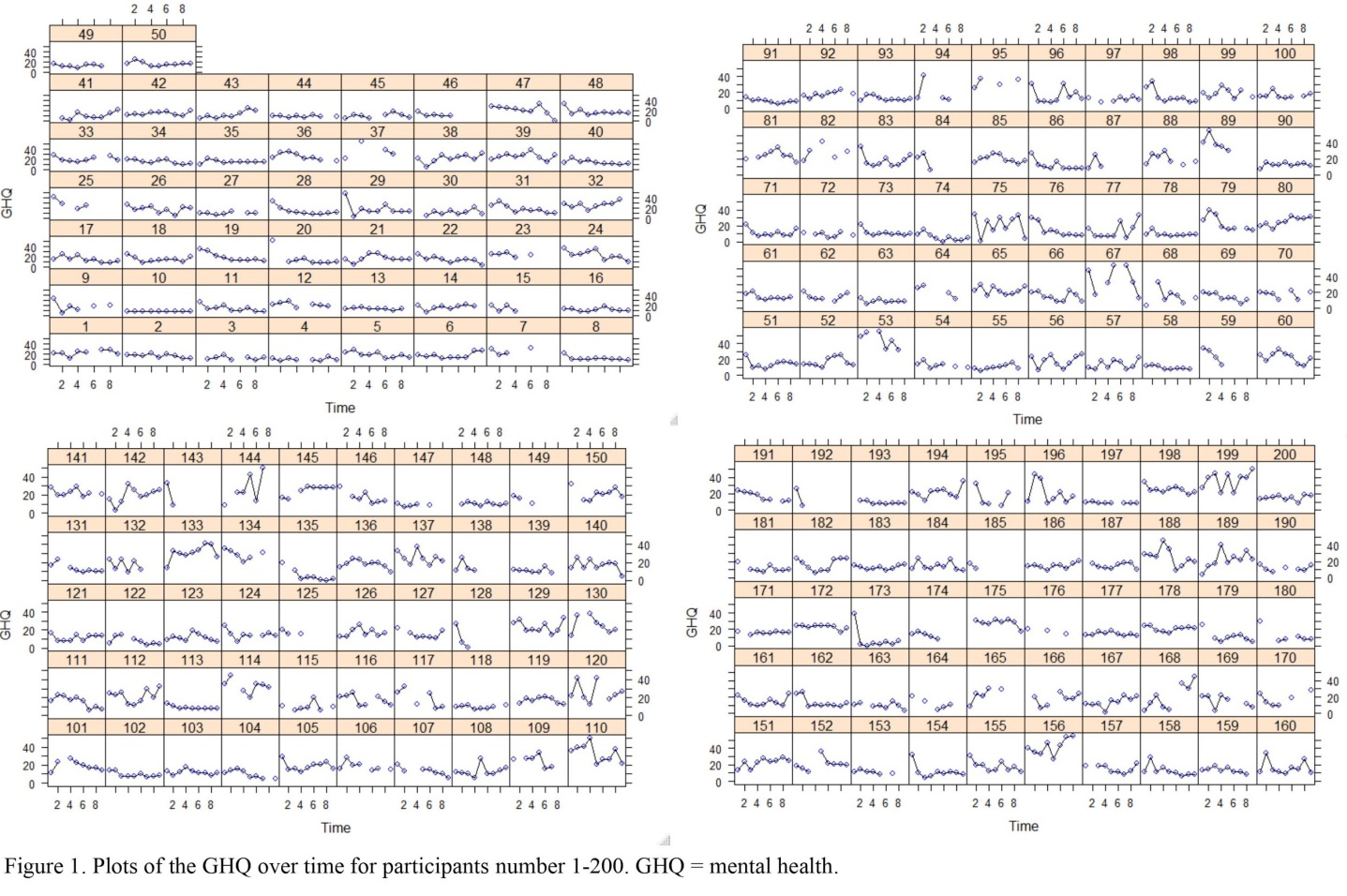


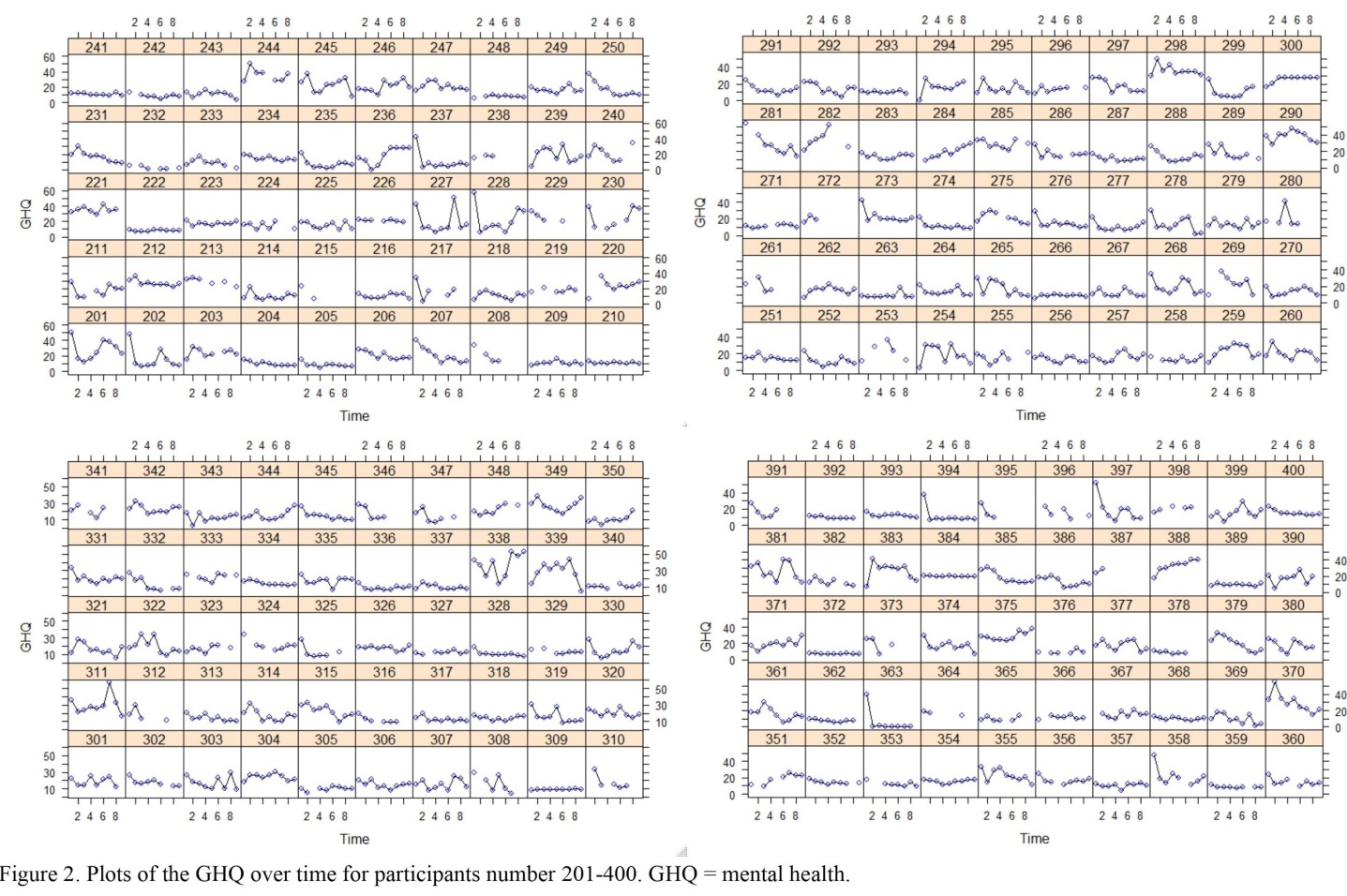

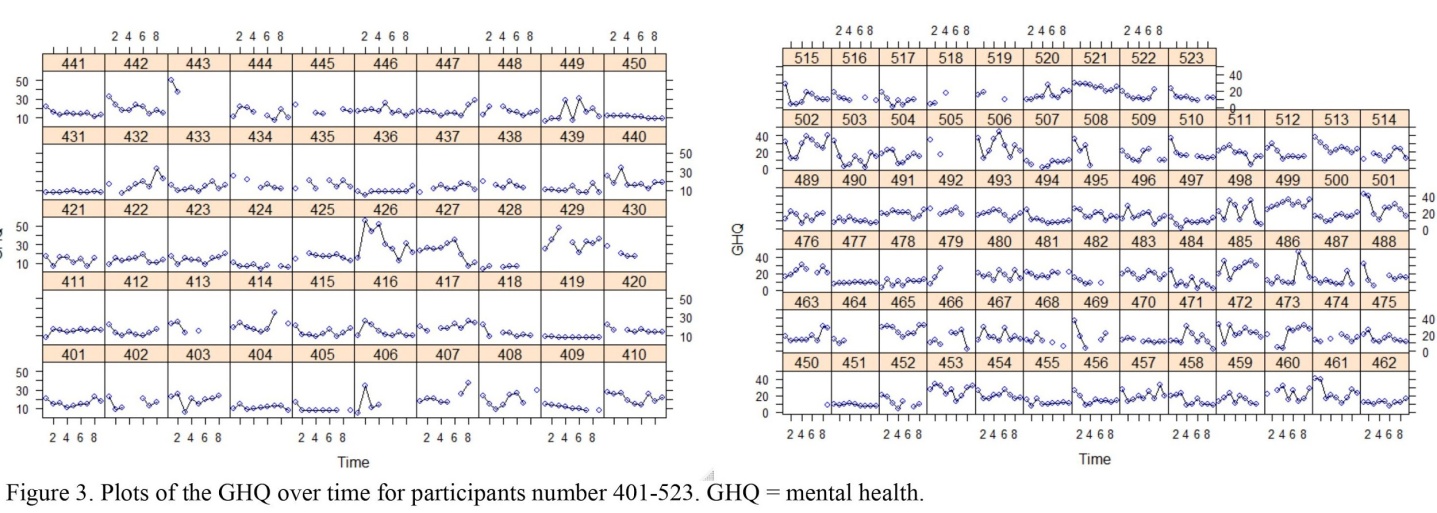


Supplementary Fig.2. Plots of mental health (GHQ) over time.

**
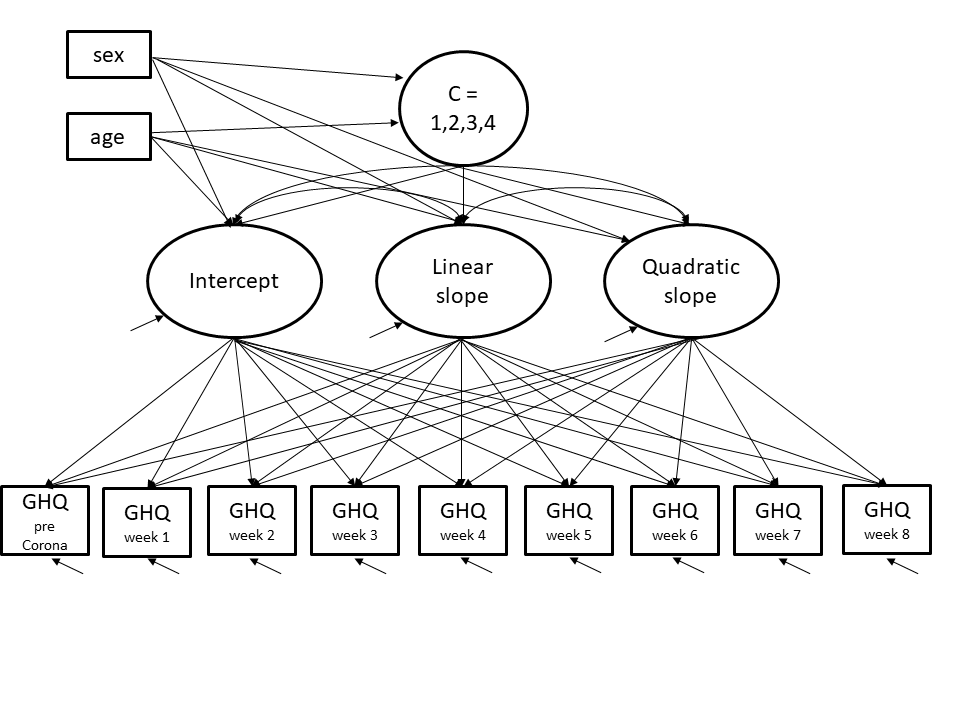
**

Supplementary Fig. 3. Latent trajectory model with one to four classes, nine observed variables (mental health, GHQ) and three growth parameters (intercept, slope, quadratic term) and two covariates (age, sex).

**Supplemental Tables**

Supplementary Table 1. *Demographics.*

| **Total: 523** | **Frequency** | **Percent** |
| --- | --- | --- |
| **Gender (m/f)** | 164/359 | 31.4/68.6 |
| **Age, mean (*SD*)** | 31.53(8.40) | 19-52 (min/max) |
| **Nationality** |  |  |
| German | 485 | 93.1 |
| European (other than German) | 25 | 4.8 |
| Asian | 8 | 1.5 |
| America(North/South) | 3 | 0.6 |
| **Relationship status** |  |  |
| Married | 119 | 23.3 |
| Widowed | 1 | 0.2 |
| Divorced/separated | 6 | 1.2 |
| Relationship, living together | 125 | 24.5 |
| Relationship, living apart | 100 | 19.6 |
| Single | 157 | 30.7 |
| Other | 3 | 0.6 |
| **Amount of people per household, including yourself** |  |  |
| 1 | 117 | 22.9 |
| 2 | 195 | 38.2 |
| 3-4 | 173 | 33.9 |
| 5-6 | 23 | 4.5 |
| more | 3 | 0.6 |
| **People in household younger than 18 years old** |  |  |
| 0 | 390 | 77.2 |
| 1 | 66 | 13.1 |
| 2 | 39 | 7.7 |
| 3 | 8 | 1.6 |
| 4 | 1 | 0.2 |
| 8 | 1 | 0.2 |
| **Profession *(multiple answers allowed)^a^*** |  |  |
| Professional training | 160 | 30.6 |
| Art, entertainment, sports, media, education, science | 112 | 21.4 |
| Health care | 108 | 20.7 |
| First aider | 9 | 1.7 |
| Military | 1 | 0.2 |
| Administration, politics, industry, cleaning, logistics | 109 | 20.9 |
| Finances and economy | 34 | 6.5 |
| Goods and services (incl. vendors and gastronomy) | 35 | 6.7 |
| Other | 53 | 10.1 |
| **Current employment *(multiple answers allowed)^a^*** |  |  |
| Full-time | 214 | 40.9 |
| Part-time | 106 | 20.3 |
| Self-employed/freelance | 26 | 5 |
| Studying/professional training full-time | 182 | 34.8 |
| Unemployed | 13 | 2.5 |
| Parental leave | 9 | 1.7 |
| Other | 29 | 5.5 |

*Note*. ^a^percentages” refer to amount of people in relation to total sample; m = male, f = female; percentages refer to valid percent

Supplementary Table 2. *Positive Appraisal Weekly Questionnaire*.

| Everyone is confronted with negative or unpleasant events from time to time and everyone reacts to them in their own personal way. In the following questions, you will be asked to state what you usually thought during the last week when a negative or unpleasant event happened to you. It is possible that none of the possible responses apply to you. | | | | |
| --- | --- | --- | --- | --- |
| Item | 0 = “not at all” | 1 = “a little” | 3 = “rather” | 3 = “very much” |
| 1) I think that I can learn something from the situation. |  |  |  |  |
| 2) I think up a plan on how I can make the best out of it. |  |  |  |  |
| 3) I think that I have to accept the situation. |  |  |  |  |
| 4) I try to distance myself from the situation and my feelings. |  |  |  |  |
| 5) I tell myself that there are worse things in life. |  |  |  |  |

Supplementary Table 3. *Questionnaires.*

| Abbr. | Questionnaire | Construct | Items | Scale | Additional information | Reference |
| --- | --- | --- | --- | --- | --- | --- |
|  | ***Mental health outcome*** | | |  |  |  |
| **GHQ** | German version of the General Health Questionnaire (GHQ-28) | Overall mental health and dysfunctions  measure of emotional distress and rates participant's subjectively reported health over the last week  items are assessed on four scales, including somatic symptoms, anxiety/sleeplessness, social dysfunction, and severe depressive symptoms | 28 | 4-point Likert scale (ranging from 0-3; 0 = least symptomatic answer, 3 = most symptomatic answer) | threshold for distress is a total sum score of 23/26^68^ | ^23,24^ |
| **PSS** | German version of the Perceived Stress Scale (PSS-10) | Negatively experienced stress during the past week | 10 | 5-point Likert scale (ranging from 1 = “never” to 5 = “very often”) | Higher scores indicate greater levels of stress | ^29^ |
| **PHQ** | Patient Health Questionnaire-4 (PHQ-4) | Core symptoms or signs of depression and anxiety over the past week | 4 | 4-point Likert scale (ranging from 0 = “not at all” to 3 = “nearly every day”). | Elevated PHQ-4 scores are not used for diagnostic purposes, but instead can be understood as an indicator for possible symptoms of anxiety and depression. | ^30^ |

Supplementary Table 3, *continued.* *Questionnaires.*

| Abbr. | Questionnaire | Construct | Items | Scale | Additional information | Reference |
| --- | --- | --- | --- | --- | --- | --- |
|  | ***Assessment of stressors*** | |  |  |  |  |
| **DH** | Chronic stressors and daily hassles with Mainz Inventory of Microstressors (MIMIS) | Assessment of stressor load in last seven days | 58 daily hassles  and  mean stressfulness of these events within the past seven days | 58 DH: ranging from 1-7 days 1-7 days  and  stressfulness: ranging from 1 = “not at all” to 5 = “very much” |  | ^31^ |
| **CE** | COVID-19 Crisis Specific Stressors Scale | COVID-19 crisis specific stressors | 34 | 4-point Likert scale (ranging from 0 = “not at all straining” to 4 = “very straining”). |  | ^28^ |
|  | ***Proposed Predictors*** | |  |  |  |  |
| **SOC** | Perceived Social Support Questionnaire (SOZU-K-10/7) | Perrceived social support | 7 | 5-point Likert scale (ranging from 1 = “not at all” to 5 = “fits exactly”) |  | ^35^ |
| **CSS** | Perceived Change in Social Support Question | Changes in social support system during the COVID-19 crisis | 1 | 5-point Likert scale (ranging from 1 = “strongly decreased” to 5 = “strongly increased”) |  | ^28^ |
| **LON** | Loneliness Scale | Loneliness | 3 | 4-point Likert scale ranging from 1 = “often” to 4 “never” | Low values on the loneliness scale indicate strong feelings of loneliness | ^32,33,34^ |

Supplementary Table 3, *continued. Questionnaires.*

| Abbr. | Questionnaire | Construct | Items | Scale | Additional information | Reference |
| --- | --- | --- | --- | --- | --- | --- |
| **PAP** | Positive Appraisal weekly Questionnaire | Positive appraisal of situations | 5 | 4-point Likert scale (ranging from 0 = “Not at all” to 3 = “Very much”). |  | Unpublished in-house developed questionnaire, see Supplementary Table 1 |
| **COPE** | Brief COPE Scale | Coping strategies individually used in the past week to deal with stress | 28 | 4-point Likert scale (ranging from 1 = “not at all” to 4 = “very much”) |  | ^36^ |
| **CERQ** | Abridged version of the Cognitive Emotion Regulation Questionnaire | Adaptive cognitive emotion regulation strategies | 12 | 5-point Likert scale (1 = “(almost) never“ to 5 = “(almost) always)” |  | ^37^ |
| **PD** | Phyiscal Distance Questionnaire | Whether phyiscal distancing recommendations were implemented in participants' daily lives | 14 | 2-point scale with the following answer options: 1 = “applied”, 2 = “not applied” and NA = “not applicable” |  | Questions were derived from recommendations on phyisical distancing from the federal center for health education ^45^ |
| **CAP** | COVID-19-related Positive AppraisalQuestionnaire | Positive appraisal specifically with regard to the COVID-19 crisis | 4 | 5-point Likert scale (ranging from 0 = “totally disagree” to 4 = “totally agree” |  | ^38^ |

|  | DH frequency pre-lockdown | |  | Averaged DH frequency for weeks 1-8 | |  |  |  |
| --- | --- | --- | --- | --- | --- | --- | --- | --- |
| Daily hassle item | *M* | *SD* |  | *M* | *SD* | *df* | *t* | *p* |
| Bad weather | 2.43 | 1.94 |  | 0.59 | 0.71 | *508* | 21.69 | **<.001** |
| Commuting | 3.18 | 2.22 |  | 1.38 | 1.73 | *508* | 16.83 | **<.001** |
| Waiting time or delay | 1.81 | 1.77 |  | 0.65 | 0.86 | *508* | 15.56 | **<.001** |
| Social obligation | 2.14 | 2.02 |  | 1.00 | 1.52 | *508* | 13.00 | **<.001** |
| Time pressure | 2.24 | 2.17 |  | 1.13 | 1.51 | *508* | 12.42 | **<.001** |
| Traffic | 0.71 | 1.30 |  | 0.05 | 0.18 | *508* | 11.71 | **<.001** |
| Own physical discomfort | 2.34 | 2.32 |  | 1.62 | 1.60 | *508* | 7.50 | **<.001** |
| Noise | 1.15 | 2.04 |  | 0.57 | 1.14 | *508* | 6.92 | **<.001** |
| High demands/high workload | 2.22 | 2.39 |  | 1.54 | 1.76 | *508* | 6.62 | **<.001** |
| Conflict or disagreement at work | 0.63 | 1.16 |  | 0.34 | 0.60 | *508* | 6.36 | **<.001** |
| Performance situation | 1.14 | 1.92 |  | 0.66 | 1.26 | *508* | 6.10 | **<.001** |
| Physical discomfort of a close person | 1.31 | 2.17 |  | 0.77 | 1.23 | *508* | 6.04 | **<.001** |

Supplementary Table 4. *Means and standard deviations of the 12 most frequent daily hassle items; comparison between pre-lockdown*

*and 8 weeks assessment.*

*Note.* All *p*-values are Bonferroni corrected *p*-values.

|  | 10 most straining daily hassles items pre-lockdown | | |  |  | 10 averaged most straining daily hassles items for weeks 1-8 | | |
| --- | --- | --- | --- | --- | --- | --- | --- | --- |
|  | Item | *M* | *SD* |  |  | Item | *M* | *SD* |
| 1 | Financial problems | 1.92 | 1.13 |  | 1 | Bad news | 1.94 | 1.15 |
| 2 | Conflicts or disagreement with close persons | 1.85 | 0.90 |  | 2 | Child care problems | 1.83 | 0.97 |
| 3 | Bad news | 1.79 | 1.19 |  | 3 | Discrimination or mobbing by another person | 1.80 | 1.30 |
| 4 | Child care problems | 1.79 | 0.96 |  | 4 | Financial problems | 1.77 | 1.01 |
| 5 | Lack of help/ support from others | 1.75 | 1.01 |  | 5 | Problem/inconvenience due to job/study/apprenticeship search | 1.71 | 1.00 |
| 6 | High demands/high workload | 1.72 | 1.06 |  | 6 | Conflicts or disagreement with close persons | 1.71 | 1.00 |
| 7 | Side effects of medications | 1.69 | 1.05 |  | 7 | Lack of help/ support from others | 1.64 | 0.95 |
| 8 | Problem/inconvenience due to job/study/apprenticeship search | 1.64 | 1.03 |  | 8 | Unsafe environment | 1.61 | 1.08 |
| 9 | Conflict or disagreement with own child/children | 1.63 | 1.02 |  | 9 | Side effects of medications | 1.58 | 1.01 |
| 10 | Conflict or disagreement at workspace | 1.62 | 1.22 |  | 10 | Performance situations | 1.58 | 1.08 |

Supplementary Table 5. *Means and standard deviations of the 10 most straining daily hassle items; comparison between pre-lockdown and during 8 weeks assessment.*

Supplementary Table 6. *Means and standard deviations of the study variables, from week one to eight.*

| Week | GHQ | | PHQ | | PSS | | Daily Hassles | | | | LON | |
| --- | --- | --- | --- | --- | --- | --- | --- | --- | --- | --- | --- | --- |
|  |  | |  | |  | | DH days | | DH burden | |  |  |
|  | *M* | *SD* | *M* | *SD* | *M* | *SD* | *M* | *SD* | *M* | *SD* | *M* | *SD* |
|  |  | |  | |  | |  |  |  |  |  |  |
| 1 | 18.42 | 9.65 | 1.92 | 2.04 | 13.07 | 6.75 | 56.27 | 25.60 | 20.98 | 14.64 | 8.28 | 2.31 |
| 2 | 16.46 | 8.45 | 1.64 | 1.84 | 11.87 | 6.42 | 46.64 | 23.05 | 16.40 | 12.54 | 8.36 | 2.26 |
| 3 | 15.74 | 8.62 | 1.66 | 1.91 | 11.22 | 6.36 | 41.90 | 21.47 | 15.03 | 11.72 | 8.26 | 2.40 |
| 4 | 16.27 | 8.17 | 1.64 | 1.85 | 11.59 | 6.42 | 42.96 | 22.41 | 15.66 | 12.97 | 8.17 | 2.35 |
| 5 | 16.28 | 7.85 | 1.61 | 1.75 | 11.41 | 6.20 | 43.07 | 22.21 | 15.79 | 12.02 | 8.33 | 2.29 |
| 6 | 16.30 | 8.89 | 1.71 | 2.07 | 11.67 | 6.44 | 42.20 | 23.51 | 15.45 | 13.30 | 8.33 | 2.36 |
| 7 | 16.41 | 8.21 | 1.65 | 1.83 | 11.61 | 6.24 | 42.40 | 23.16 | 15.48 | 13.47 | 8.29 | 2.28 |
| 8 | 15.43 | 7.95 | 1.62 | 1.87 | 11.51 | 6.22 | 41.24 | 22.35 | 14.71 | 12.41 | 8.55 | 2.33 |
| *N* | 382-482 | | 383-481 | | 386-481 | | 381-479 | | 381-476 | | 385-482 | |

*Note.* DH days: total amount of all hassles within past week multiplied by the number of days, on which the hassle occurred; DH burden: mean stressfulness over all events occurring within the past seven days.

Supplementary Table 6, *continued. Means and standard deviations of the study variables, from week one to eight.*

| Week | CAP | | Critical Events | | | | CERQ | | PAP | | CSS | | SOC | |
| --- | --- | --- | --- | --- | --- | --- | --- | --- | --- | --- | --- | --- | --- | --- |
|  |  | | CE count | | CE burden | |  | |  | |  |  |  | |
|  | *M* | *SD* | *M* | *SD* | *M* | *SD* | *M* | *SD* | *M* | *SD* | *M* | *SD* | *M* | *SD* |
|  |  | |  | | | |  | |  | |  |  |  | |
| 1 | 12.60 | 2.49 | 17.81 | 7.98 | 27.08 | 14.70 | 37.40 | 8.78 | 8.88 | 2.57 | 3.21 | 0.70 | 30.69 | 4.78 |
| 2 | 12.60 | 2.35 | 15.55 | 8.81 | 21.04 | 13.25 | 35.89 | 8.91 | 8.43 | 2.59 | 3.18 | 0.66 |  | |
| 3 | 12.36 | 2.45 | 14.06 | 8.70 | 18.19 | 12.56 | 35.80 | 9.22 | 8.21 | 2.66 | 3.20 | 0.66 |  | |
| 4 | 12.02 | 2.55 | 12.49 | 8.03 | 16.76 | 12.46 | 34.81 | 9.12 | 8.12 | 2.55 | 3.15 | 0.60 |  | |
| 5 | 12.10 | 2.46 | 11.69 | 7.52 | 16.00 | 11.95 | 34.72 | 9.38 | 7.91 | 2.68 | 3.08 | 0.65 |  | |
| 6 | 11.94 | 2.60 | 11.20 | 7.71 | 14.57 | 11.80 | 35.06 | 9.78 | 7.96 | 2.66 | 3.08 | 0.63 |  | |
| 7 | 11.76 | 2.57 | 10.63 | 7.43 | 14.10 | 12.91 | 34.18 | 9.63 | 7.62 | 2.71 | 3.05 | 0.63 |  | |
| 8 | 11.88 | 2.52 | 10.02 | 7.40 | 12.08 | 11.21 | 34.20 | 9.79 | 7.68 | 2.78 | 3.09 | 0.57 |  | |
| *N* | 384-482 | | 389-483 | | | | 384-480 | | 384-494 | | 384-482 | | 491 | |

*Note.* SOC was only assessed in week 1.

Supplementary Table 7. *Fit indices.*

| No. of classes | AIC | BIC | Sample-Size Adjusted BIC | Adj.LMR_LRT (*p*) | VLMR (*p*) | Entropy | Sample Size per Class Based on Most Likely Class Membership |
| --- | --- | --- | --- | --- | --- | --- | --- |
| *quadratic LGCM* |  |  |  |  |  |  |  |
|  | 27509.46 | 27586.13 | 27528.99 | - | - | - | 523 |
| *quadratic LCGA* |  |  |  |  |  |  |  |
| 2 | 28109.73 | 28177.88 | 28127.09 | .005 | .004 | .890 | 400/123 |
| 3 | 27762.33 | 27847.52 | 27784.03 | .443 | .436 | .838 | 309/177/37 |
| 4 | 27588.28 | 27690.51 | 27614.33 | .061 | .057 | .804 | 193/227/21/82 |
| *quadratic unconditional*  *GMM* | |  |  |  |  |  |  |
| 2 | 27420.163 | 27518.134 | 27445.126 | .058 | . 054 | .788 | 450/73 |
| 3 | 27348.070 | 27467.338 | 27378.460 | .381 | .371 | .825 | 436/44/43 |
| 4^a^ | 27317.941 | 27458.507 | 27353.758 | .561 | .558 | 0.829 | 41/419/27/36 |
| *quadratic conditional GMM* | |  |  |  |  |  |  |
| *3* | *27340.940* | 27502.804 | 27382.183 | .313 | .307 | .835 | 44/432/47 |

*Note*. AIC = Akaike information criterion; BIC = Bayesian information criterion; Adj.LMR-LRT = Adjusted Lo-Mendell-Rubin Likelihood Ratio Test; VLMR = Vuong-Lo-Mendell-Rubin Test. ^a^Inadmissible solution (i.e., negative variances).

Supplementary Table 8. *Growth parameter of the three class latent unconditional growth mixture model with class-invariant intercept variances.*

|  | Intercept (I) |  | Linear Slope (S) |  | Quadratic  Slope (Q) |  |
| --- | --- | --- | --- | --- | --- | --- |
|  | Mean | Variance | Mean | Variance | Mean | Variance |
| Delayed dysfunction | 23.57*** | 48.50** | -2.99 | 1.03 | 0.52 | 0.01 |
| Resilient | 17.97*** | 17.96*** | -1.48*** | 1.03 | 0.12*** | 0.01 |
| Recovered | 25.95*** | 39.33*** | 3.02* | 1.03 | -0.518*** | 0.01 |

*Note*. **p* < .05, ***p* < .01, ****p* <.001.

Supplementary Table 9. *The influence of sex and age on class membership and growth factors (quadratic conditional GMM).*

|  | Est. | OR |
| --- | --- | --- |
| *Between-class (multinomial regression coefficients)* | |  |
| Resilient vs Delayed dysfunction^a^ |  |  |
| sex | -0.402 | 0.669 |
| age | -0.012 | 0.988 |
| Recovered vs Delayed dysfunction^a^ |  |  |
| sex | 0.954 | 2.597 |
| age | -0.084* | 0.919 |
| Recovered vs Resilient^a^ |  |  |
| sex | 1.356* | 3.882 |
| age | -0.072** | 0.930 |
| *Regression coefficients across classes* |  |  |
| Intercept |  |  |
| Sex | 1.64* |  |
| Age | -0.02 |  |
| Slope |  |  |
| Sex | -0.47 |  |
| Age | 0.01 |  |
| Quadratic Slope |  |  |
| Sex | 0.05 |  |
| Age | 0.00 |  |

*Note*. Logistic coefficients are shown. C = Class; Est. = Estimate; OR = Odds Ratio. ^a^Is the reference class. **p* < .05, ***p* < .01, ****p* <.001.
